# Using the health belief model to assess beliefs and behaviors regarding cervical cancer screening among Saudi women: a cross-sectional observational study

**DOI:** 10.1186/s12905-018-0701-2

**Published:** 2019-01-08

**Authors:** Arwa I. Aldohaian, Sulaiman A. Alshammari, Danyah M. Arafah

**Affiliations:** Department of Family & community medicine, College of Medicine, King Saud Medical University, Riyadh, Kingdom of Saudi Arabia

**Keywords:** Cervical cancer screening, Health belief model, HPV vaccine

## Abstract

**Background:**

Cervical cancer in Saudi Arabia is ranked as the third most frequent gynecological cancer among women. The Pap smear test is a screening test that can be used as a primary prevention tool for cervical cancer, and prophylactic vaccination against HPV is also considered to be a factor in decreasing the prevalence of the disease. This study aimed to assess women’s beliefs about cervical cancer and the Pap smear test. In addition, the relationship between cervical cancer and the social and demographic characteristics was also evaluated.

**Methods:**

A descriptive cross-sectional study was performed among Saudi women living in Riyadh in 2018. Women were randomly selected, and the total sample size was 450. A predesigned self-administered questionnaire that included the Health Belief Model scale was used to collect data. Data were analyzed using SPSS 21.0. *P* values < 0.05 were considered as statistically significant in this study.

**Results:**

Among the 450 participants, the Pap smear test uptake was 26% and the HPV vaccine uptake was less than 1%. A low education level and family history for cervical cancer were significantly associated with the belief of high susceptibility for developing cervical cancer (*p* < 0.05). The seriousness of the disease was recognized by 38%, and the benefit of screening was recognized by 82% of the participants. In addition, 27% of the participants perceived barriers to obtaining a Pap smear test.

**Conclusions:**

This study showed a high level of perception regarding benefits and motivation, and a low incidence of perceived barriers among women regarding cervical cancer screening. However, these attitudinal aspects did not translate into practice, as reflected by the low uptake of the screening test. Our findings imply that concerted efforts are needed to promote cervical cancer screening programs in Saudi Arabia. In view of the planned implementation of Saudi vision 2030, which emphasizes on prevention, we recommend launching a national cervical cancer screening program, to be available and accessible to all women in primary health care centers and hospitals.

## Background

Cervical cancer is a preventable disease. It is the fourth most common cancer among women globally. The estimated number of cervical cancer cases in the world is 528,000 per year. This cancer type is also considered to be the second most common cancer in developing countries with over 400,000 cases reported yearly. Cervical cancer is the third cause of cancer-related death in developing countries (230,158 deaths per year) [1]. This means that 80% or more of the global burden of cervical cancer occurs in developing countries [2]. The explanation behind the higher mortality of cervical cancer in developing countries is that in these countries, the spending on cancer prevention programs is only approximately 5% of that spent by developed countries worldwide [3].

In Saudi Arabia, cervical cancer is the third most common gynecological malignancy in Saudi women. The incidence rate of new cases of cervical cancer was 1.9 per 100,000 in 2014 [4]. The estimated number of new cervical cancer cases and the number of deaths per year was 152 and 55, respectively [5]. However, more than 40% of women having cervical cancer are diagnosed at advanced stages in Saudi Arabia, compared to 25% in British Columbia, Canada. This high prevalence of delay in diagnosis is probably due to the lack of effective prevention and screening programs in Saudi Arabia [5, 6].

Studies have recognized a strong association between cervical cancer and human papillomavirus (HPV) serotypes 16 and 18 [7]. Almost 70% of cervical cases are caused by HPV 16 and 18 [8]. This virus affects changes in the cervical epithelium metaplasia, and such changes usually occur more rapidly during puberty [7]. The Advisory Committee on Immunization Practices in the United States of America (USA) has recommended routine HPV vaccination for girls at the age of 11 or 12 years. Vaccination may be performed at any time until the age of 26 years for those who have not been vaccinated previously or have not finished the three-dose series [9].

There are many risk factors for cervical cancer, and include the following: sexual activity at younger than 21 years of age (1.5-fold increase in risk compared to that for sexual activity initiated from 18 to 20 years of age and 2-fold increase for that initiated at less than 18 years of age), having multiple sexual partners, use of hormonal contraceptives for more than 5 years, increasing parity (three or more full-term pregnancies), smoking, and a history of sexually transmitted infections [10, 11]. The primary goal of cervical cancer prevention is to reduce the incidence by addressing the causes and the risk factors [12].

The cervical cancer screening test allows for early detection of cervical cancer. Pap smear screening, which identifies cytological abnormalities of the cervical transformation zone, has helped to reduce the incidence and mortality rates of cervical cancer by 70% in developed countries within 3 years after implementation of screening programs [13–15]. It is well known that cancer develops 10 years or more after the development of detectable precancerous lesions. Women between 30 and 40 years of age are at a higher risk of precancerous lesions [16].

Accordingly, the Saudi guideline for cervical cancer screening recommends that the universal screening strategy be followed [5]. The United States Preventive Services Task Force has recommended a Pap smear test every 3 years for women aged between 21 and 65 years. For women aged between 30 and 65 years who want to increase the length of the screening interval, a Pap smear test combined with a HPV test conducted every 5 years is recommended [17].

Unfortunately, many women remain asymptomatic until the disease has advanced, especially those women who are not sexually active. The presenting early symptoms of invasive cervical cancer are vaginal discharge, irregular bleeding, postcoital spotting, and pre- or postmenopausal spotting or bleeding. Furthermore, more advanced symptoms are urinary frequency and urgency, lower abdominal pain, severe back pain, and weight loss [18].

It is essential to study the risk factors for cervical cancer among women as well as the emotional, cognitive, and environmental aspects, which influence the women’s decision to participate in screening programs. The Health Belief Model (HBM) focuses on a person’s health-related behavior for predicting future actions [19]. The HBM has been tested, translated, and used to study women in different cultures [20].

According to this model, the decision to participate in programs designed to prevent or detect disease is determined by many factors: perceived susceptibility to the health condition, awareness of the impact of disease on their health (perceived severity), perceived benefits of undergoing screening, and perceived barriers and costs of the screening methods [19, 21].

To date, there have been no studies employing the HBM with regard to cervical cancer in Saudi Arabia. Therefore, the aim of this study is to 1) assess women’s beliefs toward cervical cancer and the Pap smear test in Riyadh, and 2) to assess women’s knowledge and beliefs regarding cervical cancer and the Pap smear test in relation to socio-demographic characteristics. The results of this study may provide a baseline assessment for future intervention programs to promote early detection and early management of cervical cancer.

## Methods

### Study design and setting

A cross-sectional study was conducted in all women who attended the gynecology outpatient clinics in Riyadh’s four main hospitals (King Khaled University, Alyamamah, King Saud Hospital, and the King Fahad Medical City) and primary care centers in Riyadh that were randomly selected by sectors as defined by the ministry of sectoral health division. Two primary care centers from each sector (North, South, Center, East, and West) were selected (for a total of 10 primary care centers).

### Sample size

We used the electronic sample size calculator, the openEpi software, to determine the required sample size. The equation used for this calculation is given below:

$$ \mathrm{n}=\left[{\mathrm{DEFF}}^{\ast}\mathrm{Np}\left(1-\mathrm{p}\right)\right]/\left[\right(\mathrm{d}\ 2/\mathrm{Z}\ 2\ 1-\upalpha /{2}^{\ast}\left(\mathrm{N}-1\right)+{\mathrm{p}}^{\ast}\left(1-\mathrm{p}\right)\Big] $$

n = required sample size

(N: Population size(for finite population correction factor or fpc) (1000000))

(p: Hypothesized % frequency of outcome factor in the population (50%+5))

(d: Confidence limits as % of 100 (absolute + 5%))

Design effect (for cluster surveys-DEFF): 1

z= confidence level at 95% (standard value of 1.96)

m = margins of error at 5% (standard value of 0.05)

sample size = 385; the expected non-response rate was set at 20%

Total sample size: 450

### Sampling technique and study subjects

The investigator collected the data from January 15, 2018 to February 30, 2018. Participants were selected randomly as follows: 150 women from the King Khalid University Hospital, 150 from the Ministry of Health Hospitals (50 from each hospital), and 150 from primary care centers (30 from each sector).

The inclusion criteria were: Saudi women (1), aged 18 years or older (2) not diagnosed with gynecological cancer, and (3) agreed to participate in the study.

### Data collection

A predesigned self-administered questionnaire was composed according to the findings from three validated published studies that were conducted in Turkey, Egypt, and Alahsa (Saudi Arabia). Furthermore, CHBM components were added to the questionnaire after obtaining permission from the copyright owner [19, 22, 23]. This self-administered questionnaire includes questions about the following topics: (1) socio-demographic characteristics; (2) knowledge about cervical cancer and about the Pap smear test; (3) HPV)-related questions; and the (4) HBM scale for cervical cancer and for the Pap smear test. The following section describes the various parts included the in the questionnaire and the topics covered in each part:

#### Part I

Socio-demographic characteristics such as age, area of residence, educational status, working status, marital status, age at marriage, duration of marriage, number of living children, usage of hormonal contraception, smoking, and family history of cervical cancer.

#### Part 2

Knowledge about cervical cancer and about the Pap smear test (yes, no, and I do not know options for being familiar with the Pap smear test; the source of information about the Pap smear test; knowledge about the Pap smear test being the main cervical cancer screening test; knowledge that cervical cancer is the most frequent cancer among women; yes and no options for whether the participant had undergone a Pap smear test; knowledge about the appropriate age at which women require a Pap smear test). Possible signs of cervical cancer were queried in 10 items (weight loss, blood in the stool or urine, vaginal bleeding after the menopause or after having sexual intercourse, pain during sexual intercourse, heavy of prolonged menstrual periods, persistent vaginal bleeding or discharge, pelvic pain and lower back pain) with “yes”, “no”, and “I do not know” options. Correct responses for possible signs were assigned a score of one point while the responses “No” or “I do not know” were assigned zero points.

The possible risk factors of cervical cancer were queried using 8 items (HPV status, smoking, weakened immunity, use of contraceptive pills, history of Chlamydia infections, marriage at a younger age, having many children, and not undergoing Pap smear tests regularly) using 5-point Likert-type scale options (strongly disagree (1 point) to strongly agree (5 points)). One item was removed from the original form from the risk factors section (having many sexual partners), since it is a sensitive question in view of the conservative nature of the Saudi society.

#### Part 3

Assessing the uptake of HPV vaccination with “yes,” “no,” and “I do not know” options. The reasons for not obtaining the vaccination and age of vaccination were also queried. The internal consistency measure (Cronbach’s alpha) of the modified instrument was 0.784 for knowledge of cervical cancer and the HPV vaccine (all items), 0.86 for the signs and symptoms section (10 items), and 0.69 for the risk factors section (eight items), as revealed by pilot testing.

#### Part 4

Using the HBM scale for cervical cancer and the Pap smear test. The scale was used based on Victoria Champion CHBM scale. The format, content, and validity of scale were tested and used in different language and culture. Permission was obtained from Champion to adapt the scale and to make the necessary changes to render them in order to be valid for both Arabic language and culture. This scale has five subscales: Perceived susceptibility to having disease was assessed by using three items “It is likely that I will get cervical cancer in the future”, “My chances of getting cervical cancer in the next few years are high”, and “I feel I will get cervical cancer sometime during my life.”; perceived seriousness of cervical cancer was assessed by seven items, e.g., “ Problems I would experience with cervical cancer would last a long time.” Perceived benefits of undergoing a Pap smear test were assessed by eight items, e.g., “Having regular Pap smear Tests will help to detect changes to the cervix before they turn into cancer.” Perceived motivation toward improving health was assessed using three items, e.g., “I eat well-balanced meals for my health.” Perceived Pap smear test barriers were evaluated using 18 items, e.g., “I am afraid to have a Pap smear Test for fear of a bad result.” All items of the subscales have the following five-point Likert-type response choices: strongly disagree (1 point), disagree (2 points), neutral (3 points), agree (4 points), and strongly agree (5 points). Each of the subscales was assessed separately, and the total score was not calculated. Subscale scores were calculated for each participant. Higher scores indicate stronger feelings about that construct. All subscales had positive responses related to the screening behavior, except for barriers which are negatively associated. In the original test, Cronbach’s alpha coefficients for the five subscales were observed to fall between 0.62 and 0.86. In this study, Cronbach’s alpha coefficients of 0.89 were observed for the five subscales.

The English questionnaires were translated by two language experts into Arabic and were back-translated to English by two different independent language experts according to Beaton-recommended guidelines [24]. This questionnaire was reviewed by two family physicians, two gynecologists, and one community professor. All the above reviewers are academic experts in their respective fields.

### Pilot study

Prior to the main study, the author conducted a pilot study with the questionnaire in 40 women to check the applicability and clarity, and to identify any difficulties with the questionnaire; the pilot study was also employed to ensure the cultural and scientific appropriateness of the instrument for the Saudi community, as well as to estimate the time needed to fill out the questionnaire. The questionnaires take approximately 15–20 min to fill out. Modification of the questionnaire was performed according to the results of the pilot study. Women who participated in the pilot study were excluded from the main study.

### Data management

Data were analyzed using the SPSS 21.0 software (SPSS Inc., IBM, USA). The socio-demographic characteristics and knowledge of cervical cancer in participating women are reported as mean, median, number, and percentage distribution, as appropriate. The average score on the HBM Scale for cervical cancer and that for the Pap test (reported as numbers, percentage, and median) were analyzed by parametric (independent sample t-test) and non-parametric tests (Kruskal Wallis test). Correlation analysis (analysis of variance test) was used to determine the relationship between socio-demographic characteristics, knowledge of cervical cancer, and the HBM scale for cervical cancer and the Pap smear test. *P* values < 0.05 were accepted as statistically significant.

## Results

Four hundred and fifty women participated in the present study. Table 1 shows that all participants in the study lived in Riyadh city. The mean age of the study participants was 32.9 ± 8.3 years. Majority of the participants (306; 68%) had a university level or higher education status. A little over half of the participants were not employed (244; 54%). The majority of participants were married (366; 81%), while the rest were unmarried (84; 19%). The mean age at marriage was 23.1 ± 4.4 years. The mean duration of marriage was 10.3 ± 8.4 years. Roughly one-third of the participants (144; 32%) had two or three children.

**Table 1:** Socio-demographic data of participants (*n* = 450)

	Number	Percent
Age		
Mean ± SD	32.9 ± 8.3	
Range (Min. – Max.)	18–57	
Level of education		
Primary + Read & write	10	2
Intermediate school	19	4
High school	115	26
University or higher	306	68
Employment status		
Working	206	46
Not working	244	54
Marital status		
Married	366	81
Unmarried^a^	84	19
Single	58	13
Divorced	22	5
Widow	4	1
Age at marriage		
18 to 29 years	325 (82.9%)	83
Mean ± SD	23. 1 ± 4.4	
Range (Min. – Max.)	12.0–38.0	
Marriage period		
Mean ± SD	10.3 ± 8.4	
Range (Min. – Max.)	0.08–37	
Number of children		
None	128	28
One child	83	19
2–3 children	144	32
4–5 children	58	13
6 or more children	37	8

*SD* standard deviation, *Min* minimum, *Max* maximum, ^a^ include (Single, Divorced, Widow)

Table 2 shows that older women believed that it is beneficial to have a Pap smear test, while women who had a low education level believed they were more susceptible to developing cervical cancer. Women who were working had more knowledge about the signs of cervical cancer. Women who had a long marriage duration were more motivated to promote self-health than did others. Women who had more children believed they were more susceptible to developing cervical cancer. Non-smoking women perceived a benefit in undergoing a cervical cancer screening test. Additionally, women who were socially interactive with health professionals, family, and friends were more aware about the Pap smear test barriers than were women who obtained their information from the media. Women who had a positive family history of cervical cancer believed they were more susceptible to developing cervical cancer.

**Table 2:** Relationship between socio-demographic characteristics, knowledge, and HBM scale for cervical cancer and the pap test

Socio-demographic characteristics	*N*	Knowledge about sing of CC (*n* = 450)	Knowledge about risk factors (*n* = 450)	Susceptibility (*n* = 450)	Seriousness (*n* = 450)	Benefits (*n* = 450)	Health Motivation (*n* = 450)	Barriers (*n* = 450)
	Total	Median (Min. – Max.)	Median (Min. – Max.)	Median (Min. – Max.)	Median (Min. – Max.)	Median (Min. – Max.)	Median (Min. – Max.)	Median (Min. – Max.)
Age								
≤ 20 years	16	2 (0–5)	19.5 (16–26)	7.5 (3–14)	20.5 (8–34)	34.5 (25–40)	11 (5–14)	54 (41–73)
21–30 years	198	3 (0–10)	21 (8–40)	7 (3–14)	21 (7–34)	35 (8–40)	12 (3–15)	51(18–74)
31–40 years	162	3 (0–10)	20 (8–40)	8 (3–15)	22 (7–35)	34 (8–40)	12 (3–15)	54 (18–85)
> 40 years	74	2.5 (0–10)	21 (8–31)	8 (3–15)	21.5 (8–35)	36 (26–40)	12 (6–15)	51.5 (28–72)
*P*-value	450	0.550	0.887	0.467	0.479	0.047	0.069	0.128
Education levels								
Primary school	10	0.5 (0–7)	21 (16–35)	9 (7–14)	25 (15–33)	37.5 (28–40)	12 (6–15)	55.5 (34–72)
Intermediate school	19	1 (0–6)	20 (14–24)	9 (3–13)	23 (8–34)	36 (22–40)	12 (3–15)	57 (33–72)
High school	115	2 (0–9)	20 (8–34)	7 (3–15)	22 (7–35)	35 (8–40)	12 (3–15)	51 (18–85)
University or higher	306	3 (0–10)	21 (8–40)	7 (3–15)	21 (7–35)	35 (8–40)	12 (3–15)	52 (18–80)
*P*-value	450	0.029	0.075	0.010	0.126	0.359	0.483	0.125
Employment status								
Working	206	3 (0–10)	21(8–40)	7.0 (3–15)	21 (7–35)	34.5 (9–40)	12 (3–15)	52 (20–81)
Not working	244	2 (0–9)	20 (8–40)	7.0 (3–15)	21 (7–35)	35 (8–40)	12 (3–15)	52 (18–85)
*P*-value	450	0.001	0.675	0.224	0.857	0.169	0.783	0.831
Marital status								
Single	58	2.5 (0–9)	21 (14–40)	7 (6–15)	21.5 (8–35)	35 (8–40)	12 (3–15)	56 (35–90)
Married	366	3 (0–10)	20 (8–40)	7 (3–15)	21 (7–35)	35 (8–40)	12 (3–15)	56 (23–90)
Divorce	22	1 (0–9)	23 (8–27)	8.5 (3–15)	21 (7–27)	34.5 (8–40)	11 (3–15)	54.5 (37–73)
Widow	4	1 (0–4)	22.5 (15–30)	6 (9–15)	19 (16–21)	34.5 (10–40)	10.5 (5–15)	52.5 (44–54)
*P*-value	450	0.317	0.219	0.498	0 .568	0 .868	0 .326	0 .610
Age when married								
Less than 18 years	33	4 (0–10)	20 (8–26)	8 (3–14)	20 (7–34)	36 (8–40)	12 (3–12)	58 (36–70)
18–29 years	325	3 (0–10)	21 (8–40)	7 (3–15)	21 (7–35)	35 (8–40)	12 (3–15)	56 (27–90)
30–40 years	34	1 (0–9)	23 (12–35)	7 (6–15)	22.5 (7–35)	33.5 (8–40)	12 (3–15)	58 (23–86)
*P*-value	392	0.708	0.056	0.057	0.422	0.052	0.977	0.792
Marriage period								
1 month − 5 years	135	2 (0–10)	21 (8–40)	7 (3–15)	20 (7–35)	34 (17–40)	11 (5–15)	53 (21–85)
> 5–10 years	108	3 (0–10)	20 (11–40)	8 (3–14)	21 (7–35)	35 (8–40)	12 (3–15)	51 (18–77)
> 10 years	149	3 (0–10)	20 (8–33)	7.6 (3–15)	22.5 (7–35)	35 (19–40)	12 (5–15)	52 (20–81)
*P*-value	392	0.703	0.378	0.148	0.387	0.055	0.005	0.626
Number of children								
None	128	2 (0–10)	21 (8–40)	7 (3–15)	22 (7–35)	34 (8–40)	12 (3–15)	55 (23–90)
One child	83	1 (0–10)	21 (12–40)	7 (3–15)	22 (7–35)	34 (8–40)	11 (3–13)	57 (37–88)
2 or 3 children	144	3 (0–10)	20 (8–40)	8 (3–15)	21 (7–35)	35 (8–40)	12 (3–15)	57 (28–90)
4 children or more	95	3 (0–10)	19.7 (8–33)	8 (3–15)	19.2 (9–35)	35.7 (8–40)	12 (3–15)	57 (27–72)
*p*-value	450	0.273	0.041	0.016	0.237	0.077	0.058	0.142
Use contraceptive								
Yes	110	3 (0–10)	20 (8–40)	8 (3–15)	21 (7–35)	35 (8–40)	12 (3–15)	57 (27–78)
No	340	2 (0–10)	21 (8–40)	7 (3–15)	21 (7–35)	35 (8–40)	12 (3–15)	55 (23–90)
P-value	450	0.383	0.573	0.629	0.912	0.425	0.156	0.481
Smoking								
Yes	12	4 (0–6)	23.5 (17–28)	9 (8–15)	15.5 (12–31)	31.5 (9–40)	11.5 (3–15)	54 (46–88)
No	438	3 (0–10)	21 (8–40)	7 (3–15)	21 (7–35)	35 (8–40)	12 (3–15)	56 (23–90)
*P*-value	450	0.947	0.237	0.488	0.538	0.027	0.129	0.744
Whether she had information about the Pap test?								
Yes	219	3 (0–10)	21 (8–40)	7 (3–15)	21 (7–34)	35 (8–40)	12 (3–15)	49 (18–81)
No	231	2 (0–10)	21 (8–40)	8 (3–15)	21 (7–35)	35 (8–40)	12 (3–15)	54 (18–85)
*P*-value	450	0.001	0.906	0.593	0.887	0.858	0.217	*P* < 0.0001
Source of the information about the Pap test is:								
Health professional	121	3 (0–10)	21 (8–30)	7 (3–13)	21 (7–34)	35 (8–40)	11 (3–15)	48 (18–81)
Family	17	5 (0–10)	18 (13–30)	7 (3–12)	20 (7–27)	35 (9–40)	12 (3–15)	42 (27–58)
Media	62	3 (0–9)	21 (13–40)	7 (3–13)	21 (10–32)	35 (24–40)	12 (5–15)	52 (37–65)
Other	19	4 (0–9)	22 (14–28)	8 (3–15)	22 (7–33)	36 (23–40)	12 (7–15)	51 (21–65)
P-value	219	0.096	0.061	0.897	0.658	0.900	0.265	0.005
Family history cervical cancer								
Yes	22	2 (0–8)	22 (8–30)	9 (4–15)	23.5 (7–35)	35 (9–40)	11.5 (3–15)	54 (40–69)
No	400	3 (0–10)	21 (8–40)	7 (3–15)	21 (7–35)	35 (8–40)	12 (3–15)	52 (18–85)
I don’t know	28	2.5 (0–8)	20.5 (15–27)	7 (3–15)	21 (8–34)	33.5 (24–40)	12 (5–15)	51 (30–62)
*P*-value	450	0.945	0.626	0.014	0.263	0.760	0.196	0.736

*HBM* Health Belief Model, *CC* Cervical Cancer

Table 3 shows that almost half of the participants (48.7%) had knowledge about the Pap smear test. The most popular sources of relevant information were health professionals (59.8%), followed by the media (23.7%). More than two-thirds (74%) of the participants had not undergone a Pap smear screening test. The mean age for undergoing a Pap smear screening test was 31.8 ± 7.8 years. A majority of the participants (192; 42.6%) selected the age range of 30–40 years, while 149 (33.1%) participants did not know the proper age for undergoing Pap smear screening.

**Table 3:** Knowledge and practice of participants regarding the cervical screening test (*n* = 450)

	Number	Percent
Do you know about the Pap test		
Yes	219	48.7
No	231	51.3
If the answer to the previous question was “yes”, please answer the following question (*n* = 219) The source of information for the Pap smear test		
Health professional	131	59.8
Family	17	7.8
Media	52	23.7
Other (friends, neighbors, etc.,)	19	8.7
The Pap smear test is the primary test used for cervical cancer screening		
Yes	110	50.2
No	25	11.4
I don’t know	84	38.4
Cervical cancer is the most common cancer in women		
Yes	63	28.8
No	66	30.1
I don’t know	90	41.1
Did you undergo a Pap test?(excluded single women *n* = 392)		
Yes	101	26
No	291	74
Appropriate age for screening for married women		
I don’t know	149	33.1
15–20	41	9.2
21–29	51	11.4
30–40	192	42.6
41–50	17	3.7
Mean ± SD	31.8 ± 7.8	
Range (Min. – Max.)	15.0–50	

*SD* standard deviation, *Min* minimum, *Max* maximum

Table 4 shows that the majority of participants (424; 94.2%) did not receive the HPV vaccine, and that less than 1% had been vaccinated (the rest did not know their vaccination status). The most popular reason for not receiving the vaccination was lack of knowledge about the vaccine (91.1%), while less than one-tenth of the participants (6.4%) cited other reasons such as fear of infection and refusal from parents or husband due to high cost. Majority of the participants did not know the appropriate age to receive the vaccination (258; 57.3%); the mean age for HPV vaccination among those who were vaccinated was 29.9 ± 8.6 years.

**Table 4:** Human papilloma virus (HPV) vaccine uptake among study participants (*n* = 450)

Have you been vaccinated against HPV?		
Yes	4	0.9%
No	424	94.2%
I don’t no	22	4.9%
If the answer to the previous question was “no,” then what was the reason for not being vaccinated?		
I don’t know anything about the vaccine	410	91.1%
Other (expensive, fear of infection as a result of this vaccination, and refusal of parents or husband)	29	6.4%
No answer	7	1.6%
Age at vaccination for HPV		
I don’t know	258	57.3%
18 years or less	13	2.9%
19–26 years	58	12.9%
27 years and more	121	26.9%
Mean ± SD	29.943 ± 8.588	
Range (Min. – Max.)	10–50	

Table 5 shows a comparison of HBM parameters with respect to different health institutions. We found no difference among participants with regard to HBM parameters pertaining to cervical cancer except for the parameter “benefits” which showed statistically significant differences among types of health institutions.

**Table 5:** Comparative cervical cancer health belief model parameter data of participants as per health institutions (*n* = 450)

	Health care centers (*n* = 150)	KKUH (*n* = 150)	MOH (*n* = 150)	*P*-value
Susceptibility (to cervical cancer)	8.0 (3.0) (3.0–13.0)	7.0 (3.0) (3.0–15)	7.5 (3.0) (3.0–15.0)	0.452
Seriousness (of cervical cancer)	21.0 (8.25) (7.0–33.0)	21.0 (8.25) (7.0–35.0)	21.0 (7.0) (7.0–35.0)	0.986
Benefits (of the Pap smear test)	34.0 (6.0) (8.0–40.0)	35.0 (6.0) (20.0–40.0)	36.0 (6.0) (8.0–40.0)	0.022
Health Motivation	12.0 (3.25) (3.0–15.0)	12.0 (4.0) (3.0–15.0)	12.0 (3.25) (3.0–15.0)	0.280
Barriers (to undergoing a Pap smear test)	52.5 (11.0) (18.0–81.0)	52.0 (12.0) (27.0–80.0)	51.0 (13.0) (18.0–85.0)	0.649

*Kruskal-Wallis test Median (Interquartile range) (minimum – maximum)

*KKUH* King Khaled University Hospitals, *MOH* Ministry of Health hospitals

Table 6 shows that 12% of the participants considered themselves to be at risk of developing cervical cancer (perceived susceptibility). Additionally, over 36% of the participants agreed to the statements related to the seriousness of the disease. Regarding perceived benefits of the Pap smear test, approximately 82% of the participants believed that undergoing regular Pap smear tests would help to find changes to the cervix before cancer develops, that regular Pap smear tests are the best way to diagnose cervical cancer at an early stage, and that cervical cancer treatment would be tolerable. In addition, almost 70% of women were motivated to promote their health (health motivation measures). Twenty-seven percent of the participants had experienced barriers to obtaining a Pap smear test. For example, 79% of participants preferred a female doctor rather than a male doctor, 40.5% of participants would feel embarrassed to lie on a gynecologic examination couch, 38% of women believed that they neglect or forget to undergo regular Pap smear tests. Additionally, 52% of the participants did not know where to go for a Pap smear test, and 42% believed that “if there is cervical cancer development in my destiny, having a Pap smear test cannot prevent it.” Furthermore, other perceived barriers included a belief that the Pap smear test takes a long time to administer (11.7%), the cost of the test is high (21.6%), lack of time to schedule a test (15%), bad manners of health professionals (12.2%), and that the procedure may be painful (21%). With regard to pain, out of 101 of women who had undergone a Pap smear test, only 29 (28.7%) women found it painful. In comparison, 54 (18%) out of 291 married women who did not undergo the test perceived that the Pap smear test involves a painful procedure.

**Table 6:** Health belief model scores of participants (single participants excluded: *N* = 392)

Variables	Agree	Neutral	Disagree
Susceptibility			
It is likely that I will get cervical cancer in the future	82	169	141
My chances of getting cervical cancer in the next few years are high	37	143	212
I feel I will get cervical cancer some time during my life	21	128	243
Average score	46.6	146.6	198.6
%	11.9	37.4	50.6
Seriousness			
The thought of cervical cancer scares me	173	90	129
When I think about cervical cancer, my heart beats faster	137	99	156
I am afraid to think about cervical cancer	205	69	118
Problems I would experience with cervical cancer would last a long time	140	129	123
Cervical cancer would threaten a relationship with my husband	176	103	113
If I had cervical cancer my whole life would change	175	101	116
If I developed cervical cancer, I would not live longer than 5 years	50	143	199
Average score	150.8	104.8	136.2
%	38.4	26.7	34.7
Benefits			
I want to discover health problems early	339	36	17
Maintaining good health is extremely important to me	344	31	17
I look for new information to improve my health	355	27	10
I feel it is important to carry out activities which will improve my health	352	31	9
Having regular Pap smear tests will help to find changes to the cervix, before they turn into cancer	325	54	13
If cervical cancer was found at a regular Pap smear test its treatment would not be so bad	283	63	46
I think that having a regular Pap smear test is the best way for cervical cancer to be diagnosed early	325	51	16
Having regular Pap smear tests will decrease my chances of dying from cervical cancer	276	77	39
Average score	325	46	20.8
%	82.8	11.7	5.3
Motivation			
I eat well-balanced meals for my health	310	64	18
I exercise at least 3 times a week for my health	263	91	38
I have regular health check-ups even when I am not sick	240	93	59
Average score	271	82.6	38.3
%	69.1	21.1	9.7
Barriers			
I am afraid to have a Pap smear test for fear of a bad result	88	98	206
I am afraid to have a Pap smear test because I don’t know what will happen	96	100	196
I don’t know where to go for a Pap smear test	203	73	116
I would be ashamed to lie on a gynecologic examination table	159	61	172
Undergoing a Pap smear test takes too much time	46	192	154
Undergoing a Pap smear test is too painful	83	188	121
Health professionals performing Pap smear tests are rude to women	48	166	178
I neglect or cannot remember to have a Pap smear test regularly	148	142	102
I have other problems in my life which are more important than having a Pap smear test	72	106	214
I am too old to have a Pap smear test regularly	33	87	272
There is no health center close to my house to have a Pap smear test	147	115	130
If there is cervical cancer development in my destiny, having a Pap smear test cannot prevent it	166	88	138
I prefer that a female doctor conducts a Pap smear test	310	58	24
I will never have a Pap smear test if I have to pay for it	85	91	216
I do not have time to get a Pap smear test	60	111	221
The Pap smear test may move the intrauterine device	35	234	123
My husband does not want me to get a Pap smear test	29	128	235
It Is difficult to get an appointment for a Pap smear test	125	123	144
Average score	107.4	120.1	164.5
%	27.4	30.6	41.9

## Discussion

This study focused on assessing health beliefs regarding cervical cancer and the Pap smear test among Saudi women living in Riyadh, and evaluated the association between these beliefs and socio-demographic characteristics. Our results showed that 26% of participants had undergone a Pap smear test, which is in agreement with data from other developing countries. The uptake of the Pap smear test was reported to be 23.8% in Kuwait [25], 21% in Jamaica [26], 21.9% in Turkey [27], 15.7% in Nepal [28], and 12% in Ghana [29]. In contrast, the Pap smear test uptake rate has been reported to be much higher in developed countries, at 93% in the USA [30], and 72% in the United Kingdom [31]. In fact, more than half of the participants in our study did not have any knowledge about the cervical cancer screening test. This lack of knowledge or incorrect information perceived by the participants could be the main reason for the low uptake rate of the Pap smear test, as has been shown in other studies [25, 28, 29, 32]. Hence, we recommend that all possible efforts should be made to increase awareness of the importance of the Pap smear test, and that the media should be recruited to help in this regard. A similar study conducted in Turkey also recommends involving the media in programs dedicated to improving cervical cancer awareness [27]. However, it is interesting to note that, in our study, the media was less preferred by women as a source of information, and more women sought such information from health professionals.

Currently, some of Saudi Arabia’s major hospitals offer the HPV vaccine based on a doctor’s prescription. Thus, primary healthcare physicians should provide more information about the HPV vaccine and should recommend timely vaccination to their patients [33]. The present study showed a low uptake of HPV vaccine in Saudi Arabia among women (1%); this uptake rate was significantly lower than that reported from other countries such as the United Kingdom (range: 44.0 to 93.4%) [34], USA (range: 56.1 to 60.4%) [35], Malaysia (77.0%) [36], and Egypt (19.9%) [19]. This may be due to the lack of a national HPV vaccination program in Saudi Arabia. In a recent cohort study conducted in Saudi Arabia, it was reported that out of 400 screened cervical specimens, 67 specimens (17%) were positive for HPV DNA. There were 291 Saudi women, only 47 out of them were positively tested for HPV. While there were 20 positive tests out of the 109 for the remaining non-Saudi women (Philippines, Jordanian, European, South Asian, Americans and Africans). Another study also from Saudi Arabia found that 31% of participants were positive for HPV DNA [37]. Further studies are needed to measure the real prevalence of HPV at the national level; thus, there is a need for implementing HPV awareness campaigns and to promote HPV vaccination programs in Saudi Arabia [37].

In the present study, women perceived themselves to have a low susceptibility to cervical cancer and that this cancer is a moderately serious disease. Hence, this belief may have contributed to the low uptake of the screening test. This finding is in concordance with a systemic review of 12 studies, which reported that the Pap smear test uptake was directly associated with higher scores of susceptibilities, seriousness, and benefits, and was indirectly associated with perceived barriers to undergoing the test [38, 39]. However, Saudi women in this study showed high scores for perceived benefits, motivation, and reported lower levels of perceived barriers to cervical cancer screening, which is in contrast with the findings of the previous systematic study. This apparent discrepancy may be attributed to the low awareness of the disease among the participating women, and because the screening test was offered only at tertiary care hospitals.

It is possible that a higher level of education and working status as seen in the present study were associated with better knowledge; these parameters were found to be statistically significant (*P* > 0.05) in our study. Along the same lines, a study conducted in the United Arab Emirates found a positive association between a high level of education or a “working” status and good knowledge about of cervical cancer [40].

However, women who had a low education level and a positive family history of cervical cancer believed that they were more susceptible to developing cervical cancer; the latter finding is in agreement with that reported by a Turkish study [27]. Another study found that women who have a positive family history of cancer are more likely to undergo a Pap smear test [41].

Further, more than one-third of women perceived the seriousness of the disease in the present study. Similarly, one-third of participants in a Vietnamese-American study also believed that cancer would threaten their relationship with their partner/husband and having cervical cancer would change their whole life. However, these beliefs did not translate into the action of undergoing a Pap smear test [42].

The present study showed that 27% of the participants perceived several barriers to undergoing a Pap smear test. Socio-cultural barriers included feeling embarrassed about having to undergo a gynecologic examination (40.5%) and not having access to a female doctor (79%). Participants may feel embarrassed about showing their private part/s to a physician during a physical examination, especially if the doctor is male. This finding is in line with a study conducted among Egyptian women (who shared similar Arabic and Islamic culture as Saudi women) which showed that 76.9% of women prefer a female doctor to perform the Pap smear procedure [19]. However, a study of Vietnamese-American women showed that women felt uncomfortable to undergo Pap smear screening performed by a male doctor (38.8%) [42]. Modesty and embarrassment were reported frequently as barriers for cervical cancer screening programs among Arab Muslim women in the USA [43].

False interpretation of Islamic beliefs led to 42% of women to believe that “if cervical cancer development is part of my destiny, having a Pap smear test cannot prevent it”; however, the Islamic faith encourages Muslims to perform self-care, seek treatment, and to provide health support [43]. It was narrated that the Prophet (PBUH): said: “Seek treatment, O slaves of Allah! For Allah does not create any disease but he also creates with it the cure, except for old age.” [44].

Healthcare organizations play an important role in popularizing and administering the Pap smear test. This is reflected in our study, since 52% of participants did not know where to go for a Pap smear test; this finding is in agreement with that reported by a similar study conducted in Turkey. The investigators found that the Pap smear test uptake was four times lower if the women were not aware of where the test was offered than if they were aware of this information. Therefore, cervical cancer screening programs should provide information not only regarding the benefits and the procedure, but also provide adequate information of where it is offered [45].

Many studies have shown that financial concerns may act as barriers to undergoing a Pap smear test if the women have to pay for the test. More than half of the women in two other similar studies believed it was too expensive to have a Pap smear test; however, only 21% of participants in our study considered the cost to be a barrier. This finding may be explained by the fact that the majority of women in our study had free access to hospitals or had medical insurance coverage, and that the income of the Saudi population is relatively high [42, 45].

The fear of pain involved in the Pap smear test procedure may prevent some women from undergoing the test. Therefore, explaining the procedure may reduce anxiety and improve the uptake in women. Fortunately, in our study, the percentage of Saudi women who had experienced pain during the test (among those who had undergone a Pap smear test) was low, at 28.7%; this figure is lower than that reported by a similar study conducted in Turkey (59%). The Turkish study found that fear of pain among women made them four times less likely to undergo a Pap smear test [45].

There are several limitations to this study. The women who participated in the study were selected from Riyadh city, and were highly educated. Therefore, the results may not be generalizable to all Saudi women. This cross-sectional study was conducted using a self-administered questionnaire, and recall bias cannot be ruled out due to such a study design.

## Conclusions

This study showed a high incidence of perceived benefits and motivation, and low incidence of perceived barriers among women with regard to cervical cancer screening. However, the data also showed that these perceived benefits and motivation did not translate to practice, and only a small percentage of women underwent the screening test. Furthermore, there is no current national program for cervical cancer screening in Saudi Arabia. Following the implementation of the new Saudi vision 2030 and health system reform, which emphasizes on the importance of prevention, we recommend launching a national cervical cancer screening program, which is available and accessible to all women at primary health care centers and hospitals. Such a program should be designed to improve awareness, to overcome any perceived barriers to screening, and hopefully to increase the uptake of screening. These measures may promote earlier detection of cancer among women and contribute to lowering the mortality rates associated with cervical cancer.

## Abbreviations

HBM: Health belief model
HPV: Human papillomavirus
USA: United States of America
